# Dispersal Strategies, Few Dominating or Many Coexisting: The Effect of Environmental Spatial Structure and Multiple Sources of Mortality

**DOI:** 10.1371/journal.pone.0034733

**Published:** 2012-04-06

**Authors:** Lucie Büchi, Séverine Vuilleumier

**Affiliations:** 1 Department of Ecology and Evolution, University of Lausanne, Lausanne, Switzerland; 2 Agroscope Changins-Wädenswil Research Station ACW, Nyon, Switzerland; National Institute of Water & Atmospheric Research, New Zealand

## Abstract

Interspecific competition, life history traits, environmental heterogeneity and spatial structure as well as disturbance are known to impact the successful dispersal strategies in metacommunities. However, studies on the direction of impact of those factors on dispersal have yielded contradictory results and often considered only few competing dispersal strategies at the same time. We used a unifying modeling approach to contrast the combined effects of species traits (adult survival, specialization), environmental heterogeneity and structure (spatial autocorrelation, habitat availability) and disturbance on the selected, maintained and coexisting dispersal strategies in heterogeneous metacommunities. Using a negative exponential dispersal kernel, we allowed for variation of both species dispersal distance and dispersal rate. We showed that strong disturbance promotes species with high dispersal abilities, while low local adult survival and habitat availability select against them. Spatial autocorrelation favors species with higher dispersal ability when adult survival and disturbance rate are low, and selects against them in the opposite situation. Interestingly, several dispersal strategies coexist when disturbance and adult survival act in opposition, as for example when strong disturbance regime favors species with high dispersal abilities while low adult survival selects species with low dispersal. Our results unify apparently contradictory previous results and demonstrate that spatial structure, disturbance and adult survival determine the success and diversity of coexisting dispersal strategies in competing metacommunities.

## Introduction

Dispersal is an ubiquitous phenomenon which affects the dynamics, ecology, genetics and evolution of natural populations [1]–[4]. The mechanisms leading to and maintaining dispersal have been extensively studied and can have genetic or ecological bases. Genetic drivers are mainly the avoidance of competition with kin [5]–[8] (but see also [9]), the maintenance of genetic variability and avoidance of inbreeding depression [10]–[13]. Ecological drivers are environmental spatiotemporal variability and stochasticity, i.e. habitat heterogeneity, availability and distribution [6], [14]–[16] and extinction-recolonization processes [4], [17]–[18].

Genetic drivers mainly select for dispersal, except when migrants disrupt local adaptation [19]–[21]. Ecological drivers can have more ambiguous impacts on dispersal. Habitat heterogeneity and low availability induce a cost for dispersing individuals, as they face the risk to end up in unsuitable habitats [22]–[23], and thus select against dispersers. However, this cost depends also on the habitat spatial autocorrelation. High spatial autocorrelation can favor dispersal, as clustering tends to bring together favorable habitats, and so locally decreases dispersal cost [24]–[26]. But, it also has been shown that, in clustered habitats, reduced dispersal rate and distance can be selected due to close availability of favorable habitats [27]–[29]. Environmental stochasticity and disturbance, causing local species extinction, are known to select for dispersal. Indeed, local extinction tends to eliminate philopatric individuals, and creates settlement opportunity for dispersers [6], [15], [17], [30]–[31]. But recently, studies have suggested that dispersal rate is not always monotonically increasing with extinction rate [18], [32]–[34]. When extinction is strong, populations remain under carrying capacity and allow local recruitment of individuals, thus favoring some philopatry. Due to their potential opposite effects, how genetic and ecological factors interact to either select for or against dispersal remain unclear in numerous situations.

Although dispersal has been mostly studied at the population level, dispersal is also known to strongly impact community and metacommunity properties, such as composition, dynamics and persistence [35]–[36]. Dispersal also drives species coexistence, for example through competition-colonization trade-offs [37] or neutral processes [38], and thus shape community diversity (see [39] for a synthesis of dispersal-diversity relationship). The observation of natural systems at the community level reveals a huge diversity of forms and expressions of dispersal, and not a unique optimal strategy, contrary to what is often predicted by models. The diversity of dispersal strategies is expected to be shaped by species specific characteristics and interspecific competition, which can balance the relative benefits and costs of dispersing, in interaction with the environment. In particular, the adult survival rate might modify the intensity of competition between juveniles and adults and thus may change the benefits of dispersal. Also, species specialization determines the amount of habitat available, as well as the habitat spatial distribution experienced by the species. These influence in turn the probability of ending in an unsuitable habitat, which could potentially affect dispersal behaviour.

Environmental heterogeneity and stochasticity as well as species life history traits are thus recognized as important determinant factors for the characteristics and diversity of coexisting dispersal strategies. However, to date, few investigations have been done to understand the maintenance of dispersal strategies taking into account the combined impact of these factors. To address these issues, we use a spatially explicit metacommunity model of species competing for space within a heterogeneous environment. With this model we quantify the combined influence of spatial autocorrelation, habitat availability, stochastic disturbance and species traits (adult survival rate and specialization) on the dispersal strategies. More specifically we investigate (i) how these factors influence the most successful dispersal strategies in the metacommunity, and (ii) which conditions maintain multiple distinct dispersal strategies. The answers to those questions give new insights on the persistence, coexistence and diversity of species with various dispersal strategies, in heterogeneous and stochastic environments.

## Methods

To investigate which dispersal strategies are selected in a competing metacommunity, we used a spatially explicit metacommunity model developed by Büchi et al. [40]. Here, the metacommunity is composed by species displaying a large diversity of dispersal strategies, and competing for space. We varied the environmental conditions of the metacommunity (spatial autocorrelation and disturbance regime) and we assessed the persistence of the species in the metacommunity.

**Figure 1:**
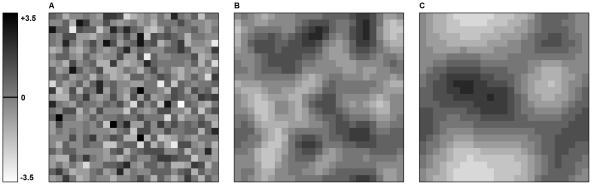
Environmental spatial structure for three degrees of spatial autocorrelation α. (a) α = 0. (b) α = 5. (c) α = 10. The landscape size is 25 cells by 25 cells. Dark cells represent high environmental values E_i_ and light cells low values.

### Model Description

Environment is modeled by a grid landscape composed of discrete, homogeneous, habitat cells (Figure 1). Each cell is characterized by an environmental value E_i_ (e.g. temperature, humidity), which determines species fecundity (as described below). This environmental value can vary from one cell to another, the landscapes generated being thus heterogeneous. The spatial distribution of the environmental values can display various degrees of spatial autocorrelation α. α is the autocorrelation range, and represents the distance above which the correlation between the environmental values of two cells drops below 0.5. The landscape average environmental value is the same across all values of α as the distribution of the environmental values follows a gaussian function with a mean of zero and standard deviation of one.

Additionally, a carrying capacity K (set here to 100) is assigned to each landscape cell. It determines the maximum number of local resident individuals. Local communities are linked by species dispersal, thus forming a metacommunity. The size of the simulated landscapes is 25×25 cells. Periodic boundary conditions were used to avoid edge effects.

Metacommunity dynamics proceeds in discrete time steps. Each step is composed of four sequential phases: 1. reproduction, 2. adult mortality and disturbance, 3. juvenile dispersal and 4. competition for space.

1. Reproduction occurs simultaneously in each cell. Fecundity R_s_ is modeled with a gaussian function that takes into account the deviation of the local environmental value E_i_ from the species niche optimum µ_s_, and the niche breadth σ_s_ of the species. This function also characterizes the specialization of the species.



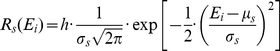
(1)Where h is a scaling factor transforming the reproductive effort into an effective fecundity (h is set to ten in this study).

2. Individual mortality can occur through two processes. First each adult can die, after reproduction, according to its mortality rate 1−ψ_s_, ψ_s_ being the adult survival probability. Thus, when adult survival is greater than zero, generations are overlapping. Second, disturbance can cause local community extinction (all individuals die, including juveniles). At each time step, a proportion T of the metacommunity (proportion of disturbed cells, cells are randomly drawn) is driven to extinction, through local community extinction.

**Figure 2:**
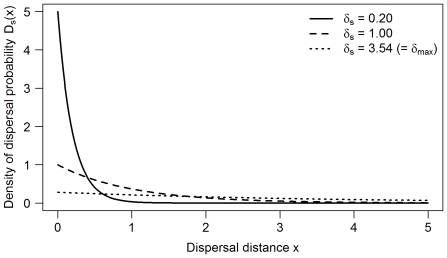
Dispersal kernel for three different species dispersal abilities δ_s_. δ_s_ = 0.20 (plain line), δ_s_ = 1.00 (dashed line) and δ_s_ = 3.54 ( = δ_max_, maximal value in this study) (dotted line).

3. A dispersal kernel D_s_(x) determines the probability for a juvenile to disperse at a distance x from its birth cell (Figure 2). This probability declines here as a negative exponential function of the distance x.



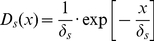
(2)The shape of the kernel depends on the mean dispersal ability of the species δ_s_. δ_s_ ranged from 0 to δ_max_, which is set to one tenth of the diagonal of the landscape. Once the dispersal distance is determined, the dispersal direction is drawn randomly from a uniform distribution.

4. Competition occurs after dispersal, when juveniles compete to settle in each local community. Only the space not occupied by resident adults (K−N_adults_) can be colonized. Each juvenile has the same probability to settle, which depends on the number of competing juveniles and on the amount of space available. If the latter is higher than the number of competing juveniles, it results in a local community under carrying capacity K. Adults remain unaffected by the competition between juveniles. After this stage, all the juveniles that succeed in settling become adults.

**Table 1:** Parameter values used for the simulations.

Parameters	Symbols	Phases	Values
Niche optimum	µ_s_	Reproduction	0
Niche breadth	σ_s_	Reproduction	0.05; 0.5
Dispersal ability	δ_s_	Dispersal	0−1 [step: 0.01] * δ_max_
Survival rate	ψ_s_	Mortality	1°; 0.95; 0.9; 0.75; 0.5; 0
External disturbance	T	Mortality	0; 0.005; 0.01; 0.1; 0.25∶0.5
Spatial autocorrelation	α		0; 5; 10

°only when T>0.

### Simulation Setup

To assess the maintenance and success of species dispersal strategies, we considered pools of 101 species which differed by their dispersal ability δ_s_ (δ_s_ ranged from 0 to δ_max_ ( = 3.54) in steps of 0.01*δ_max_, leading to 101 different species) (Table 1). A dispersal value of 0 means that juveniles are completely philopatric, while a value of 3.54 (δ_max_) corresponds to a species whose 95% of the juveniles disperse out of their natal cell. All traits except dispersal ability δ_s_ had identical values within a pool of species.

We studied the effect of environmental spatial structure by comparing species persistence on landscapes with different levels of spatial autocorrelation, α = 0 (unstructured landscapes), α = 5 (slightly structured landscapes) and α = 10 (highly structured landscapes) (Figure 1, Table 1).

The impact of generation overlap, and hence of the intensity of competition, was investigated by assigning different rates of adult survival ψ_s_ (0, 0.5, 0.75, 0.9, 0.95, 1) to the species pools, covering the range from annual species (ψ_s_ = 0) to long-lived species (ψ_s_>0.75) (Table 1).

We applied six disturbance rates T (0, 0.005, 0.01, 0.1, 0.25 0.5, proportion of disturbed cells, Table 1) to analyze its impact on the persistence of various dispersal strategies.

In addition, to investigate the influence of habitat specialization, and hence of habitat availability, on dispersal strategies, we considered successively generalist (σ_s_ = 0.5) and specialist species (σ_s_ = 0.05). All species in all pools have a niche optimum µ_s_ equal to 0 (Table 1).

Simulations were run for all the possible combinations of these four parameters (spatial autocorrelation, adult survival rate, disturbance rate, species specialization) in order to assess their single and combined effects on the dispersal strategies maintained.

Metacommunity dynamics was simulated for 5000 time steps during which some species went extinct and others persisted. This number of time steps guarantees stable conditions for all cases investigated (results not shown). We ran 50 replicates for each simulation, with a newly generated landscape for each replicate. At the beginning of each simulation, for each cell, individuals were randomly drawn from the pool of species (with replacement) until carrying capacity was reached.

**Figure 3:**
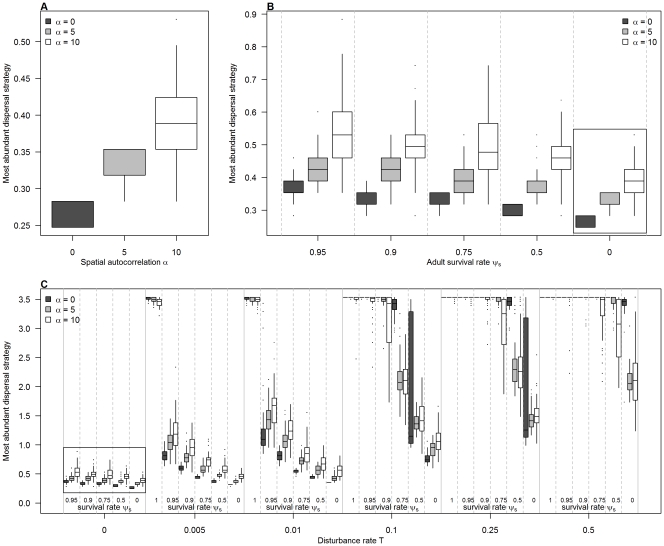
Dominant dispersal strategies for the generalist species (σ_s_ = 0.5). Most abundant dispersal strategy as a function of spatial autocorrelation α, adult survival rate ψ_s_, and disturbance rate T. (a) Influence of spatial autocorrelation when ψ_s_ = 0 and T = 0. (b) Influence of spatial autocorrelation and survival rate when T = 0. (c) Influence of spatial autocorrelation, adult survival and disturbance rate. Each box represents the distribution of the n = 50 replicates. Results for the specialist species are presented in Figure S1.

At the end of each simulation, we recorded the dispersal ability and abundance of all the surviving species. We also determined the probability of persistence of each species by computing the proportion of replicates in which the species survived. We extracted the dispersal ability of the species with the highest abundance to determine the most successful dispersal strategy in each simulation. We compared these strategies to assess the influence of spatial structure, adult survival, global disturbance, and species specialization. We then looked at the abundance distribution of all the dispersal strategies to reveal the potential coexistence of multiple dispersal strategies. Simulation outputs were analyzed using the software R 2.10.1 [41].

## Results

### Most Abundant Dispersal Strategies

Except in some cases with complex coexistence of multiple dispersal strategies (see below), the most abundant and successful dispersal strategies of the metacommunity were easily individuated. Spatial autocorrelation, adult survival, disturbance rate as well as species specialization strongly influenced the most abundant strategy in each simulation.

In the metacommunity, the most abundant dispersal strategy was affected by the spatial autocorrelation of the landscape, for both generalist and specialist species (Figure 3 and Figure S1). When species were annual and there was no disturbance (ψ_s_ = 0, T = 0), dispersal increased with spatial autocorrelation (Figure 3A). A positive relationship between dispersal and spatial autocorrelation was also observed for higher survival rates, in the case with no disturbance (T = 0) (Figure 3B). However, the effect of spatial structure was not consistent throughout all simulations, and varied according to adult survival and specialization when disturbance occurred (T>0) (Figure 3C and Figure S1). For generalist species, in the cases where the disturbance rate was intermediate (T = 0.005, T = 0.01 and T = 0.1), dispersal was positively linked to spatial autocorrelation for low to medium values of survival, while a negative relationship was observed for high values of survival (Figure 3C). At high disturbance rates (T = 0.25 and T = 0.5), the dispersal of the most abundant species always decreased with spatial autocorrelation.

**Figure 4:**
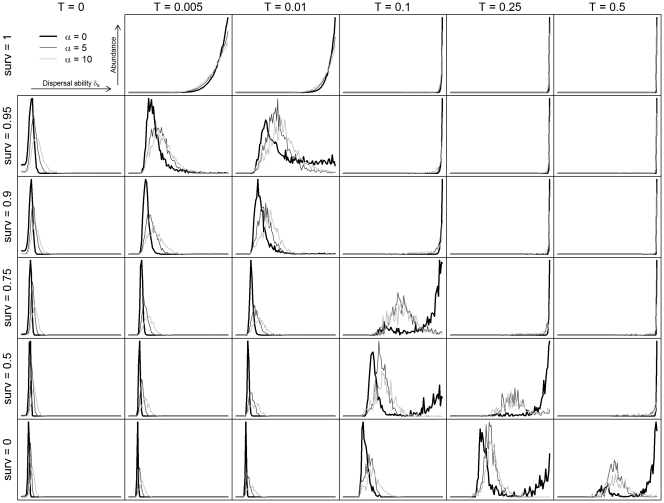
Abundance of all the dispersal strategies for the generalist species. Mean abundances of the 101 generalist species (σ_s_ = 0.5), computed on the n = 50 replicates, as a function of species dispersal ability, across the various values of adult survival rate ψ_s_ and disturbance rate T. Thick black line: α = 0; thin black line: α = 5; grey line: α = 10. Results for the specialist species are presented in Figure S2.

**Figure 5:**
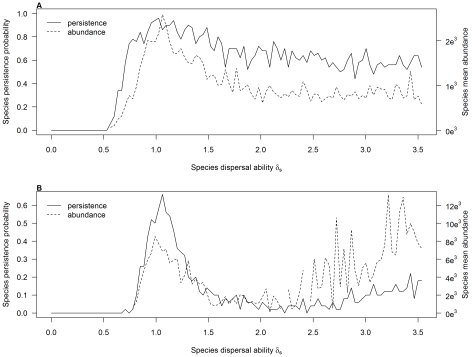
Species persistence and mean abundance as a function of their dispersal ability. On the left y-axis (continuous line) is represented the species persistence probability (proportion of replicates in which the species has survived) while the right y-axis (dashed line) shows the species mean abundances (computed only on the replicates in which the species has survived). (a) Generalist species (σ_s_ = 0.5) with very high adult survival rate (ψ_s_ = 0.95), in the presence of a low disturbance rate (T = 0.01) in an uncorrelated environment (α = 0). (b) Generalist species (σ_s_ = 0.5) with high adult survival rate (ψ_s_ = 0.5), in the presence of a medium disturbance rate (T = 0.1) in an uncorrelated environment (α = 0).

Interestingly, it appeared that positive relationships between dispersal distance and spatial autocorrelation occurred when the overall dispersal was low, whereas negative relationships occurred when dispersal was high (Figure 3C). This was visible for both generalists and specialists, although the switch between positive and negative relationships did not appear at the same values of dispersal (Figure 3C and Figure S1C).

Adult survival and disturbance rate had also a strong impact on the most successful dispersal strategies for both specialist and generalist species (Figure 3 and Figure S1), even though dispersal was generally lower for the specialist species. A low rate of local adult survival favored species with reduced dispersal ability (Figure 3B), while global disturbance had an opposite effect, strong disturbance rate selecting for high dispersal abilities (Figure 3C). Thus the highest dispersal values were obtained when survival was maximal (ψ_s_ = 1) and the disturbance rate was the strongest (T = 0.5), whereas the lowest dispersal values were obtained for annual species (ψ_s_ = 0), when no external disturbance occurred (T = 0). Between these two extremes cases, the most abundant dispersal strategies decreased from high to low values, with well observable intermediate values (Figure 3).

### Distribution of Dispersal Strategies

The distribution of the coexisting dispersal strategies depended on the adult survival rate and the disturbance regime considered. A clear dominance of one dispersal strategy, coexisting with very few other similar dispersal strategies was observed in most cases (Figure 4). However, when adult survival and disturbance rate acted in opposition on dispersal (e.g. when a strong disturbance regime favored species with high dispersal abilities while low adult survival selected species with low dispersal), a high number of distinct dispersal strategies coexisted (Figure 4). In this situation, two patterns could emerge (Figure 5). The first was composed by a dominant dispersal strategy with high persistence probability and abundance that coexisted with several other strategies, with lower probability of persistence and abundance (Figure 5A). In the second case, two groups of species with very distinct dispersal strategies (intermediate versus high dispersal) coexisted together (Figure 5B). One group (with intermediate dispersal strategies) was composed of species persisting with high probability but exhibiting medium abundances, while the other group (with high dispersal strategies) was composed by species with lower persistence probability but higher abundances.

The results were consistently similar for generalist and specialist species, although the combinations of disturbance rate and adult survival leading to the coexistence of multiple dispersal strategies differed slightly (Figure 4 and Figure S2). Higher disturbance and survival rate were necessary to maintain multiple strategies in specialist species compared to generalist ones.

## Discussion

Given the high diversity of dispersal strategies in nature, and the importance of dispersal for species survival, the evolution and maintenance of dispersal has been a long-standing object of investigations. Several studies have shown the important role for dispersal strategies of spatial and temporal heterogeneity [6], [14]–[17], [31], of the degree of competition experienced by the individuals [5], [7], [15], [31]–[32], [42]–[43], as well as of the amount and spatial repartition of available habitats [24], [29], [44]–[45].

Here, we present how spatial environmental heterogeneity and autocorrelation, disturbance, and species traits such as adult survival rate and specialization, impact the successful dispersal strategies of species competing in a metacommunity. Then, we document the conditions for which several dispersal strategies coexist within a metacommunity. Finally, we discuss some model assumptions and future issues.

### Environmental Spatial Autocorrelation

We demonstrated that environmental spatial autocorrelation strongly impacts the dispersal strategies that are maintained in a metacommunity. Depending on the adult survival rate and disturbance regime, spatial autocorrelation can either inflate or reduce dispersal. This can be explained by the relationship between the scale of the environmental correlation and the scale at which dispersal events occur. When local adult survival and disturbance rate are low, thus favoring localized dispersal, spatial autocorrelation, by grouping together suitable habitats, decreases dispersal cost and favors an increase of dispersal [26]. A decrease of dispersal occurs in the opposite situation, when adult survival and disturbance rate are high and promote high dispersal. In this situation, the spatial autocorrelation of suitable habitats inflates the probability that juveniles disperse out of the habitat clusters. Dispersal becomes costly and species with lower dispersal abilities are favored [29], [44].

These findings can help to understand apparently contradictory results on the effects of spatial structure on dispersal. While several studies showed a decrease of species dispersal in structured environments [27]–[29], [45]–[46], others found that spatial structure tends to select for species with high dispersal abilities [24]–[25]. However, in these studies, the scale at which dispersal occurs strongly differs. In the former studies, dispersal was modeled at global scale, either through global dispersal or with a continuous dispersal kernel, allowing for large dispersal events in the environment. Their results are in agreement with our study when high dispersal abilities are maintained, that is, when disturbance and adult survival rates are high. On the opposite, the latter studies used a nearest-neighbour dispersal model, thus allowing only very spatially limited dispersal. Their findings are in agreement with our results when reduced dispersal is favored, that is, in situations where both disturbance and adult survival rate are low. As suggested by recent studies [47]–[49], our results confirm that the direction of the selection of dispersal strategies depends strongly on the scale at which dispersal occurs, in interaction with the environmental spatial structure.

The impact of environmental spatial autocorrelation also depends on the level of species specialization. For the specialist species, an increase in dispersal with environmental autocorrelation is observed only in the absence of global disturbance, when very low dispersal is favored. In contrast, for generalist species, an increase in dispersal with environmental autocorrelation is observed in a larger range of situations. This relates to differences in habitat availability. For specialist species, the amount of suitable habitat is much lower compared to generalist species. Thus, even in strongly autocorrelated landscapes, the size of the specialist habitat clusters remains relatively small, and the probability for juveniles to disperse out of the clusters is high. For these reasons, a reduction of dispersal is favored for specialist species much more often than for generalist species.

### Disturbance and Adult Survival Rate

In the absence of disturbance (T = 0), the dispersal ability of the successful species is overall small. In this situation, the cost of dispersal is important, as the probability for dispersers to reach an unfavorable habitat is high and therefore dispersal is selected against [22]–[23]. As disturbance rate increases, so do the extinction risk of philopatric species. This, combined with the creation of new empty habitats, strongly favors species with large dispersal abilities [15], [17]. Our results show that this process is strongly enhanced when adult survival rate is high (generations are overlapping). Indeed, with high adult survival, local competition is important and local recruitment of juveniles is scarce. Successful species are the ones which disperse and settle into new empty habitats created by global disturbance [15], [31]–[32], [34]. A higher adult mortality increases the possibility for juveniles to establish in their natal habitat, and thus selects for lower dispersal abilities.

Local intrinsic mortality and external disturbance are thus two forces acting in opposition on dispersal, and strongly influencing the most abundant dispersal strategies in the metacommunity. In addition, the opposite effect of these two forces leads to interesting results in terms of the coexistence of multiple dispersal strategies (see section below).

Dispersal of both generalist and specialist species are influenced by local mortality rate and external disturbance. However, surviving dispersal strategies were overall lower for specialist species than for generalists, and the number of surviving species was much larger for generalist species. This difference arises from the decreased number of suitable habitats available for specialist species compared to generalist species. When habitat availability decreases, dispersal is selected against as the probability for dispersing individuals to reach an unsuitable habitat inflates drastically [24], [45].

### Coexistence of Distinct Dispersal Strategies

The coexistence of multiple dispersal strategies is achieved when local mortality and disturbance rates act in opposition on dispersal, i.e. when one favors dispersal while the other selects against it. Local mortality affects the whole metacommunity identically, and controls, in a homogeneous manner, for the number of empty spots in each local community. High mortality rates thus induce a uniform decrease in the saturation level of the metacommunity. This allows for more local recruitment and consequently favors species with reduced dispersal abilities [15], [31]. In contrast, stochastic disturbance affects localized fractions of the metacommunity. This creates strong heterogeneity in local density that promotes species with high dispersal abilities [31]–[32]. Thus, multiple and distinct dispersal strategies can coexist when the combination of survival and disturbance rate allows enough local recruitment for species with low dispersal ability to survive, and the creation of enough new empty habitats to maintain higher dispersal strategies. This mechanism is similar to the one described in Massol et al. [50], who showed that patch size heterogeneity induces disruptive selection on dispersal. Here, the combinations of local mortality and disturbance rate allowing multiple strategies to coexist also depend on habitat availability and spatial structure. This is evidenced by the differences observed in the distribution of dispersal strategies between specialists and generalists on the one hand, and between different environmental spatial autocorrelation on the other hand.

Previous studies have shown that dispersal strategies can coexist depending on the combination of the different forces acting on dispersal [16], [21], [27], [34], [50]–[56]. But most of the studies focused on the coexistence of few dispersal strategies at the same time. Here we showed that, while some parameter combinations favor a unique optimal dispersal strategy, interestingly others lead to the coexistence of a high number of distinct dispersal strategies. The selection of one or few dispersal strategies appears when the environmental conditions favor species with either very low or very high dispersal abilities. Numerous distinct dispersal strategies coexist when environmental conditions correspond to the transition between these low and high dispersal cases. Our results thus show that the number and type of dispersal strategies maintained in a metacommunity is shaped by the complex interactions between the sources of species mortality (disturbance and survival rate) and spatial environmental factors (heterogeneity and autocorrelation).

### Model Assumptions and Future Issues

To investigate the coexistence of dispersal strategies within a competing metacommunity, we followed the approach used for example by Kallimanis et al. [45] and Devictor and Robert [57]. This approach starts with a large diversity of strategies with different traits (here dispersal ability), competing together at the same time. The whole system then evolves progressively through the selection of the most successful strategies. Another approach, commonly used in population genetics, is to consider trait evolution by mutation-selection processes [32], [42], [47], which allows the successive emergence of new strategies, competing with the already established ones. The two approaches thus differ in the number and in the variability of strategies competing at the same time. Further investigations are needed to contrast the results obtained by these two approaches and might be promising to study evolving metacommunity [58]–[59].

To understand the evolution of dispersal, many studies focused on dispersal rate only, neglecting dispersal distance (but see for example [26], [28]–[29], [42], [45], [46], [60]). In contrast, we used here a dispersal kernel allowing varying dispersal distance, and not only dispersal rate. We chose the commonly assumed negative exponential kernel [3], [28], [42], [45], which has demonstrated good adequacy with numerous empirical data [61]–[62]. Given the high sensitivity of our results to dispersal pattern, we recommend that future investigations should always consider dispersal distance and avoid focusing only on dispersal rate. An even more accurate understanding of dispersal evolution is expected to be obtained using more complex dispersal functions, for example “fat-tailed” dispersal kernel allowing long-distance dispersal events [63]–[64], or functions allowing independent tuning of dispersal rate and distance, and of short- and long- distance dispersal [26], [47], [65]. These complex dispersal functions deserve deeper investigations and should be incorporated in future studies on dispersal in metacommunities.

Given the species traits and the life cycle considered, as well as the other assumptions on the dispersal process (sessile adults and passive juvenile dispersal), our model is well representative of plant species, for example in grassland metacommunities. However, our results on the impact of spatial structure and disturbance on the favoured dispersal strategies are expected to follow similar trends under other life cycles.

To allow for a better comprehension of the complex interaction between species dispersal strategies and environmental spatial structure, availability and disturbance, we focused here on the effect of dispersal alone. Nevertheless, others factors are known to impact dispersal and should be considered for future investigations. For example, additional dispersal costs such as mortality during dispersal [1], [8], [60], [66], or various levels of disturbance aggregation [45], [67], could have been added in the model. More importantly, we believe that further investigations should integrate correlations between species traits. Indeed, dispersal is often involved in trade-offs with other traits such as fecundity or competitiveness [68].

The relationship between species dispersal strategies and species coexistence, at both community and metacommunity scales, might also reveal interesting mechanisms of maintenance of diversity. In addition, our results point to the presence of interactions between dispersal and specialization (see also [21]), which are also likely to influence species diversity at multiple scales. These aspects should be the object of future investigations.

### Conclusions

We demonstrated a complex and interesting impact of spatial autocorrelation on the most successful dispersal strategies in a metacommunity, which depends also on the intensity of disturbance and adult survival, and on the amount of habitat available. We also showed that, depending on the strength of the forces acting, a few number of similar dispersal strategies, or a high number of species with distinct strategies could coexist together. A large diversity of dispersal strategies are maintained when local recruitment (driven by local adult mortality) and colonization of empty habitats (driven by stochastic disturbance) are both occurring. This might reflects what is observed in natural metacommunities, where many different forces act and interact to allow numerous dispersal strategies to coexist.

These results are of crucial importance as, with the raising pressure on natural habitats, dispersal is expected to play a more and more important role in species persistence and evolution. In particular, the current increase in disturbance rate and the degradation of habitats, reducing spatial autocorrelation, are expected to disfavour species with low dispersal ability and specialized habits, leading to a functional homogenization of natural communities.

## Supporting Information

Figure S1 **Dominant dispersal strategies for the specialist species (σ_s_ = 0.05).** Most abundant dispersal strategy as a function of spatial autocorrelation α, adult survival rate ψ_s_, and disturbance rate T. (a) Influence of spatial autocorrelation when ψ_s_ = 0 and T = 0. (b) Influence of spatial autocorrelation and survival rate when T = 0. (c) Influence of spatial autocorrelation, adult survival and disturbance rate. Each box represents the distribution of the n = 50 replicates.
(TIF)

Figure S2 **Abundance of all the dispersal strategies for the specialist species.** Mean abundances of the 101 specialist species (σ_s_ = 0.05), computed on the n = 50 replicates, as a function of species dispersal ability, across the various values of adult survival rate ψ_s_ and disturbance rate T. Thick black line: α = 0; thin black line: α = 5; grey line: α = 10.
(TIF)
