# Supplementary figures and images for: Dispersal Strategies, Few Dominating or Many Coexisting: The Effect of Environmental Spatial Structure and Multiple Sources of Mortality
Source: PLoS One. 2012 Apr 6;7(4):e34733. doi: 10.1371/journal.pone.0034733 (PMC3321035; doi:10.1371/journal.pone.0034733)

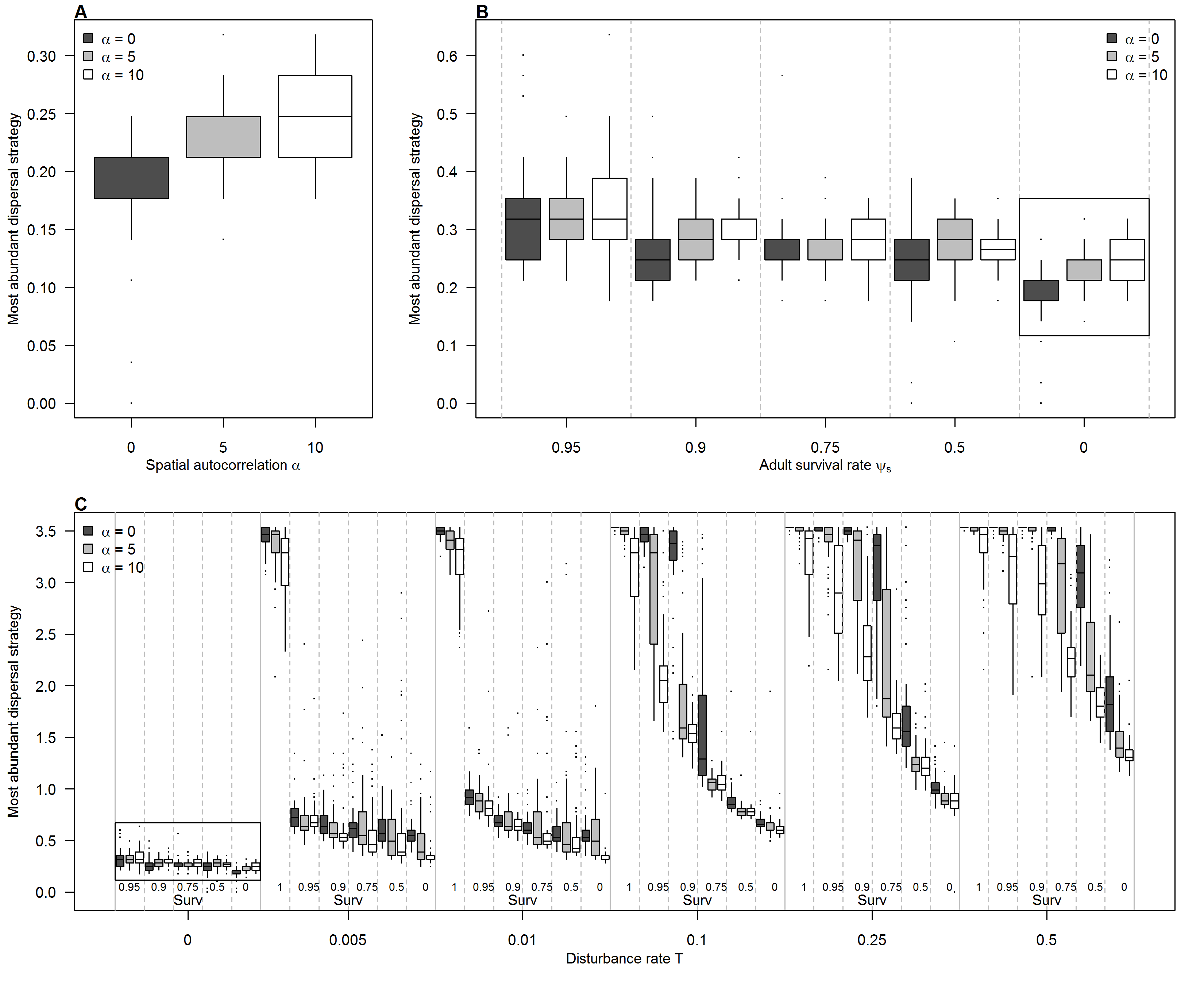

Supplement: Figure S1 — Dominant dispersal strategies for the specialist species (σs = 0.05). Most abundant dispersal strategy as a function of spatial autocorrelation α, adult survival rate ψs, and disturbance rate T. (a) Influence of spatial autocorrelation when ψs = 0 and T = 0. (b) Influence of spatial autocorrelation and survival rate when T = 0. (c) Influence of spatial autocorrelation, adult survival and disturbance rate. Each box represents the distribution of the n = 50 replicates. (TIF) [file pone.0034733.s001.tif]

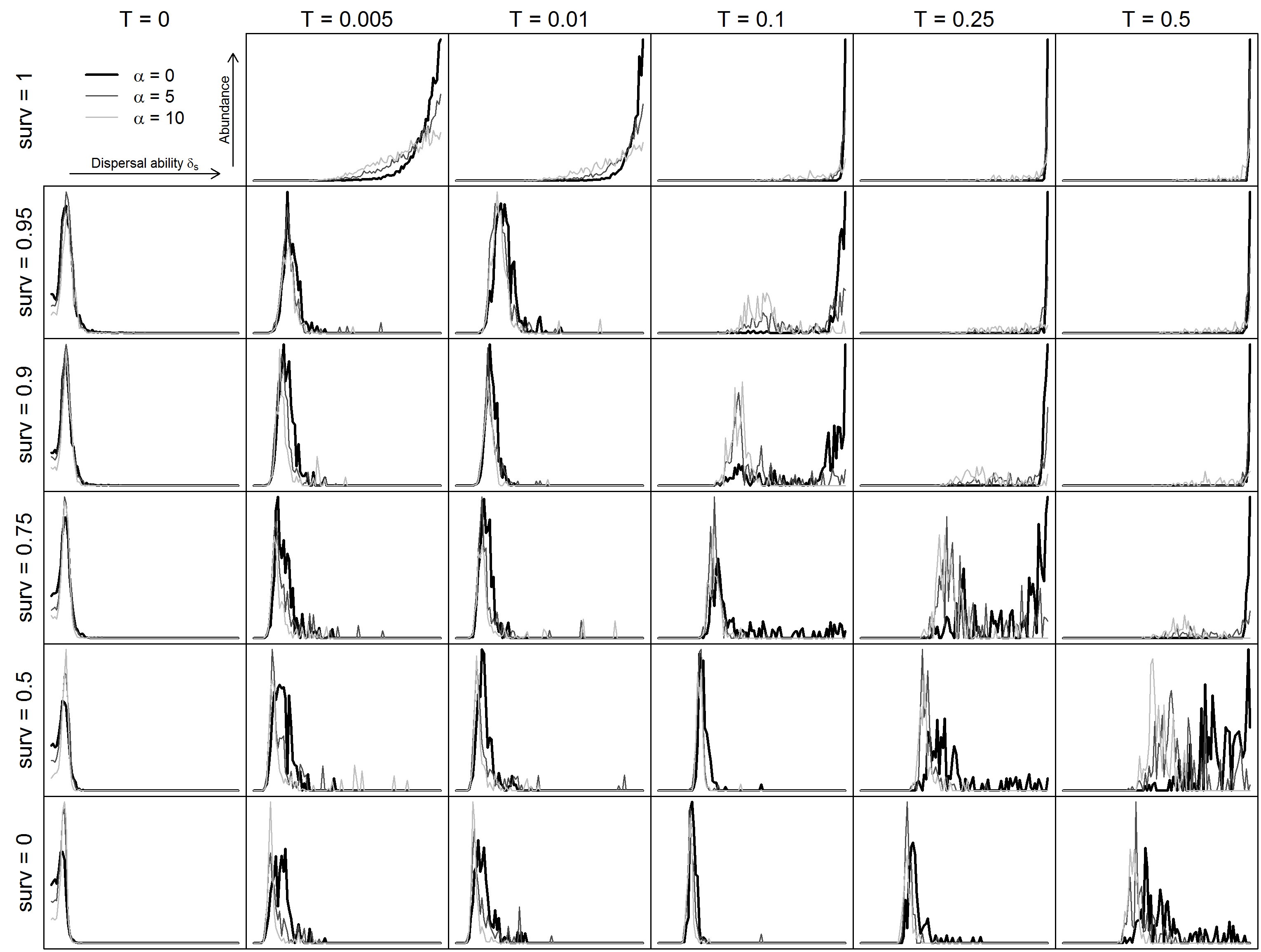

Supplement: Figure S2 — Abundance of all the dispersal strategies for the specialist species. Mean abundances of the 101 specialist species (σs = 0.05), computed on the n = 50 replicates, as a function of species dispersal ability, across the various values of adult survival rate ψs and disturbance rate T. Thick black line: α = 0; thin black line: α = 5; grey line: α = 10. (TIF) [file pone.0034733.s002.tif]
